# Lycopene-mediated mitigation of cadmium-induced nephrotoxicity in broilers is associated with restoring mitochondrial homeostasis

**DOI:** 10.1016/j.psj.2026.107251

**Published:** 2026-06-10

**Authors:** Haodong Wang, Zhi Lu, Gongyin Chen, Xuebing Wang, Xu Yang, Cong Zhang

**Affiliations:** a College of Veterinary Medicine, Henan Agricultural University, Zhengzhou, Henan 450002, China; b Department of Psychological and Cognitive Sciences, Tsinghua University, Beijing 100084, China; c Pet Science College, Henan Vocational College of Agriculture, Zhengzhou, Henan 450002, China

**Keywords:** Cadmium, Lycopene, Mitochondrial quality control, Broiler, Kidney

## Abstract

Cadmium (Cd) is a ubiquitous environmental pollutant with high bioaccumulation potential, primarily targeting the kidney by inducing oxidative stress and mitochondrial dysfunction. This study aimed to investigate the protective effects and underlying molecular mechanisms of lycopene (LYC) against Cd-induced nephrotoxicity in broilers. In this study, Arbor Acres broilers were administered Cd-containing diets with or without LYC supplementation for 42 days and then assessed growth performance, serum biochemistry, and renal morphology. The results showed that LYC significantly reversed Cd-induced growth inhibition and renal histopathological damage. Further analysis revealed that the alleviating effect of LYC on Cd-driven renal impairment was linked to the regulation of the mitochondrial biogenesis, mitochondrial dynamics, and mitophagy. These results suggested that LYC mitigated Cd-induced nephrotoxicity in broilers were associated with the regulating of mitochondrial quality control-related factors and the restoration of mitochondrial homeostasis. Thereby, this study further demonstrates the mechanism by which LYC alleviates Cd-induced nephrotoxicity.

## Graphical abstract

**Figure:**
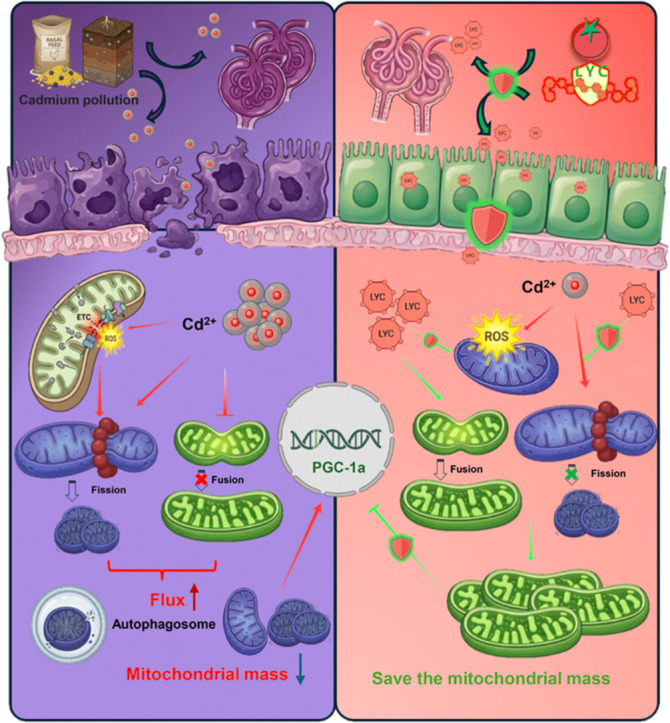

## Introduction

Cadmium (Cd), a highly toxic heavy metal pollutant, has emerged as a global threat to animal health and food safety owing to its environmental persistence, bioaccumulation, and widespread environmental distribution. Industrial wastewater discharge, excessive application of Cd-contaminated phosphate fertilizers, and improper disposal of electronic waste have led to Cd accumulation in soil, water, and feed chains (Houessionon et al., 2021; Suciu et al., 2022; Oladimeji et al., 2024). Through the “environment-feed-animal” transmission pathway, Cd poses significant challenges to livestock production, particularly in poultry farming. Notably, Cd is a ubiquitous environmental pollutant that has been classified as a Group 1 human carcinogen by the International Agency for Research on Cancer (IARC) and is associated with increased risks of chronic kidney disease, cardiovascular disease, and all-cause mortality even at relatively low levels of exposure, indicating that environmental Cd exposure exerts a non-negligible impact on human health. As one of the most widely consumed poultry species worldwide, broilers are uniquely susceptible to Cd bioaccumulation (Aljohani, 2023; Ahmad et al., 2024). The rapid growth and high metabolic turnover of broilers accelerate the bioaccumulation of Cd from contaminated feed and water into the animal products (e.g., meat and egg), further escalating risks to human health through the food chain. Additionally, Cd exposure in broilers has been shown to impair growth performance, reduce reproductive performance, cause immune dysfunction and multiple organ impairment (Aljohani, 2023). Hence, revealing the toxic mechanism of Cd has significant practical significance, which provides a scientific basis for public health risk assessment.

The kidney serves as a primary site for both Cd accumulation and excretion, rendering it especially susceptible to Cd-induced toxicity (Heshmati and Salaramoli, 2015). The epidemiological and experimental studies have been recognized that renal injury is widely link to Cd exposure (Dong et al., 2025). The structural injuries lead to glomerular swelling and extensive necrosis and shedding of renal tubular epithelial cells in a concentration-dependent manner. The structural injuries lead to impaired renal excretory function, as evidenced by elevated serum creatinine (SCr) and blood urea nitrogen (BUN). The progressive damage ultimately results in tubulointerstitial fibrosis and chronic kidney disease, contributing to increased mortality and reduced production efficiency in poultry (Lee et al., 2024). The nephrotoxicity of Cd is generally attributed to oxidative stress, inflammatory signaling, and apoptosis, which collectively impair tubular epithelial integrity and progressively compromise renal function (Ansari et al., 2017; Li et al., 2021; Yan and Allen, 2021). These processes are interrelated and often amplify one another, producing the histological and functional hallmarks of Cd-induced renal injury. However, these mechanisms do not fully delineate the complex pathogenesis of the nephrotoxicity mechanism of Cd, and further exploration of related mechanisms is still needed. Given the vital role of the kidney as a target of Cd toxicity and in systemic homeostasis, investigating the mechanisms of nephrotoxicity and protective interventions is essential for understanding and mitigating the hazard of Cd in broiler production. Such efforts are urgent priorities for ensuring sustainable animal husbandry and providing a theoretical basis for public health risk assessment.

Mitochondria are double-membrane-bound organelles, often referred to as the “powerhouses of the cell” due to their central role in generating adenosine triphosphate (ATP) through oxidative phosphorylation (Hall and Unwin, 2007). Mitochondrial dysfunction serves as a central mediator of Cd-induced renal injury. Renal cells, particularly those within the proximal tubules, rely heavily on mitochondrial ATP to power active solute transport; consequently, these cells are characterized by a high mitochondrial density and robust respiratory capacity. Furthermore, this high energetic demand renders renal mitochondria particularly vulnerable to toxic, ischemic and metabolic insults and makes mitochondrial dysfunction an early and sensitive indicator of renal stress. Hence, mitochondrial damage is recognized as a common and universal phenotype across various forms of kidney injury and kidney disease (Tang et al., 2021; Guo et al., 2024).

In response to mitochondrial insults and to ensure bioenergetic stability, cells employ a multi-dimensional mitochondrial quality control (MQC) system, which governs the complete mitochondrial life cycle and is orchestrated by three pivotal, interlinked processes: mitochondrial biogenesis, mitochondrial dynamics, and mitophagy. Specifically, in avian models, Cd disrupts the renal MQC system by impairing the mitochondrial unfolded protein response (UPRmt) and Nrf2-dependent antioxidant defenses, while simultaneously dysregulating mitochondrial biogenesis and dynamics. These disturbances culminate in structural cristae damage, loss of mitochondrial membrane potential (ΔΨm), elevated mitochondrial reactive oxygen species (ROS) production, and the subsequent release of cytochrome C (Cyt C) (Ge et al., 2019). Aberrant mitophagy exacerbates mitochondrial network fragmentation and energy deficits in renal tubular epithelial cells, while Cyt C release activates the caspase cascade (Wang et al., 2004). Collectively, impairment in MQC system further facilitate excessive apoptosis or necrosis of renal tubular epithelial cells and ultimately impair renal structure and function. Given the critical role of MQC system disorder in Cd-induced nephrotoxicity, preserving mitochondrial homeostasis and reducing mitochondrial damage represent a promising therapeutic strategy to mitigate Cd nephrotoxicity. Furthermore, screening for compounds that protect MQC system could identify candidate drugs to treat Cd-induced renal toxicity.

Lycopene (LYC) is a naturally occurring, acyclic carotenoid primarily found in red fruits and vegetables such as tomatoes, watermelon, pink grapefruit, and guava. LYC is a highly unsaturated tetraterpene carotenoid characterized by a long-conjugated double-bond system, which underlies its strong singlet-oxygen and free-radical quenching capacity (Dai et al., 2022). LYC is abundant in commonly consumed foods such as tomatoes and watermelons, and its lipophilicity and oral bioavailability can be improved by dietary lipids or food processing, addressing the limitations of many existing Cd detoxification strategies (e.g., low bioavailability and poor targeting). Importantly, toxicology reviews report a favorable safety profile at commonly used dietary and experimental doses, which is a critical prerequisite for its potential application as a therapeutic agent (Arballo et al., 2021).

LYC has demonstrated significant renoprotective potential, primarily by counteracting Cd-induced oxidative stress and inflammation, while concurrently bolstering endogenous antioxidant capacity and safeguarding mitochondrial integrity (Pan et al., 2025; Song et al., 2026). In a lipopolysaccharide-induced acute kidney injury model, LYC pretreatment improved renal function and alleviated oxidative stress and mitigated mitochondrial dysfunction (Salari et al., 2022). Accumulating evidence further indicates that LYC preserves mitochondrial integrity by modulating PINK1/Parkin-dependent mitophagy and balancing fusion-fission dynamics such as Mfn1/2 and Drp1, thus preventing mitochondrial loss and bioenergetic failure under toxic stress (Park et al., 2021; Cai et al., 2023; Zheng et al., 2026). Additionally, LYC has also been reported to preferentially accumulate in mitochondrial fractions, further confirming that mitochondria are key target organelles for LYC to play a protective role (Bradley et al., 2023). Together, these reports support the renoprotective benefits of LYC in exerting antioxidant, anti-inflammatory, and mitochondrial-stabilizing effects. Therefore, it is proposed that LYC may mitigate mitochondrial damage by maintaining the MQC system, thereby alleviating Cd-induced nephrotoxicity.

In this study, dietary LYC significantly alleviated Cd-induced renal injury in broilers. LYC regulated mitochondrial dynamics-related factors and mitophagy-related markers to inhibit mitochondrial damage,and maintain mitochondrial homeostasis. This study aimed to elucidate the protective effects and underlying molecular mechanisms of LYC against Cd nephrotoxicity, providing a theoretical foundation for developing mitochondria-targeted detoxification agents in poultry production.

## Materials and methods

### Experimental protocol and sample collection

A total of 80 one-day-old Arbor Acres (AA) broilers were purchased from Luohe Ririhong Poultry Industry. As shown in Table 1, these broilers were randomly divided into 4 groups. Based on previous reports that the oral LD50 of Cd in broilers was 218.44 mg/kg body weight and our preliminary observations, 140 mg/kg Cd was selected as the challenge dose (approximately 3/5 of the LD50) (Zhang et al., 2020). The LYC dosage (400 mg/kg) was determined by integrating national regulatory requirements with preliminary experimental data. Adhering to the GB/T 40942-2021 standard, we utilized the maximum tolerance limit to optimize the protective potential. Previous trials have confirmed that this dosage is physiologically safe and well-tolerated in broilers, ensuring no negative impact on growth performance or overall health (Sahin et al., 2016). After 42 days of feeding, broilers were fasted for 12 h and euthanized according to institutional guidelines. Blood was collected and centrifuged to separate serum. Kidneys were removed and photographed immediately. One portion of each kidney was fixed in 4% paraformaldehyde for histology. Additional portions were snap-frozen in liquid nitrogen and stored at −80°C for molecular analyses (qRT–PCR and Western blot). The study was conducted according to the guidelines of Institutional Animal Care and Use Committee (IACUC) at the Henan Agricultural University (Approval number: HNND2024053101). Unless otherwise stated, all molecular assays were performed using three independent biological replicates per group, each derived from a different animal.

**Table 1:** Animal groups.

Groups	Number	Dietary Treatment
Control group	20	basal diet
Cd group	20	basal diet supplemented with 140 mg/kg CdCl₂
LYC+Cd group	20	basal diet supplemented with 400 mg/kg LYC and 140 mg/kg CdCl₂
LYC group	20	basal diet supplemented with 400 mg/kg LYC

### Renal histopathology and injury scoring

Kidney tissues were fixed in 4% paraformaldehyde for H&E staining. Tissue fixation, trimming, dehydration, paraffin embedding, and sectioning were performed by Servicebio Technology Co., Ltd. Subsequently, paraffin sections were deparaffinized and rehydrated. The sections were then stained with hematoxylin and eosin, followed by dehydration and mounting. Finally, images were captured using a digital slide scanning system for observing renal pathological changes.

H&E-stained kidney sections were examined by two observers who were blinded to group allocation. Glomerular lesions were scored semi-quantitatively on a 0–4 scale (0 = no lesion; 1 = 0–25% area affected; 2 = 25–50%; 3 = 50–75%; 4 = 75–100%). At least 10 glomeruli per animal were evaluated. Tubular injury was scored on a 0–4 scale based on the percentage of damaged tubules in fields of the outer medulla at 200 × (0 = normal; 1 < 5%; 2 = 5–25%; 3 = 25–75%; 4 > 75%). Mean scores per animal were used for group comparisons (representative micrographs were taken; scale bar = 50 μm).

### Determination of renal function

The SCr and BUN were measured using a Beckman AU680 automated clinical chemistry analyzer to assess kidney function.

### Assessment of oxidative stress status

Detection of oxidative stress markers was performed using commercial kits provided by Nanjing Jiancheng. Tissue suspensions were prepared via mechanical dissociation, and then the T-AOC (A015-1), SOD (A001-2), CAT (A007-2-1) and MDA (A003-1) assays were performed using relevant assay kits according to the manufacturer’s instructions.

### Detection of mitochondrial ROS and ΔΨm

Broiler kidneys were collected on ice and mechanically dissociated. Following enzymatic digestion, cell suspensions were filtered through a 40-µm strainer into complete medium supplemented with 10% FBS. Cell concentration was determined via hemocytometer after trypan blue staining and subsequently adjusted to 1.0 × 10⁷ cells/mL.

Mitochondrial ROS was measured using commercial kits (S0061S, Beyotime, Shanghai, China) following the manufacturer’s instructions. Prepared tissue cell suspension was washed two times with RPMI 1640 medium and then incubated with MitoSOX working solution at 37 °C for 30 min in the dark, washed two times with PBS, and imaged immediately in 48-well plates.

JC-1 staining was used to detect the ΔΨm by using commercial kits (C2003S, Beyotime, Shanghai, China) following the manufacturer’s instructions. Prepared tissue cell suspension was washed two times with complete medium (with 10% FBS) and then added to JC-1 working solution and incubated in a cell culture incubator at 37 °C for 20 min, washed twice with JC-1 buffer and imaged immediately in a 48-well plate.

### Quantitative real-time PCR (qRT–PCR) analysis

Total RNA was extracted from frozen kidney tissue using RNA extraction solution (G3013, Servicebio, Wuhan, China) according to the manufacturer’s instructions. RNA concentration and purity were assessed spectrophotometrically. Complementary DNA (cDNA) was synthesized from equal amounts of total RNA using the HiScript® II Q RT SuperMix for qPCR (+gDNA wiper) kit (G3337, Servicebio, Wuhan, China). The qRT–PCR was performed using the ChamQ Universal SYBR qPCR Master Mix (G3323, Servicebio, Wuhan, China) on the qTOWER® (analytikjena, Germany) system. Each sample was run in technical triplicate. The relative mRNA expression was calculated by the 2^(-ΔΔCt) method and normalized to the internal reference gene β-actin. The primer sequences are shown in Table S1.

### Western blotting analysis

Total protein from kidney was extracted with RIPA lysis buffer (G2002, Servicebio, Wuhan, China). Mitochondrial fractions were isolated from kidney tissue using a commercial mitochondrial isolation kit (C0010, Applygen, Beijing, China) following the manufacturer’s instructions. The isolated mitochondria were subsequently lysed in RIPA buffer to obtain mitochondrial proteins. During mitochondrial isolation, the resulting supernatant was designated as the cytosolic protein fraction and used for the detection of cytosolic Cyt C content. The protein concentration was determined using the Coomassie G-250 (PC0015, Solarbio, Beijing, China) assay with BSA (PC0001, Solarbio, Beijing, China) as the protein standard. Equal amounts of protein were separated by SDS-PAGE and transferred to polyvinylidene difluoride (PVDF) membranes. Membranes were blocked with 5% non-fat milk and incubated with primary antibodies overnight at 4°C. After washing, membranes were incubated with appropriate horseradish peroxidase (HRP)-conjugated secondary antibodies. Protein bands were visualized using an enhanced chemiluminescence (ECL) system and imaged. Densitometry was performed using ImageJ analysis software. The primary antibodies are shown in Table S2.

### Statistical analysis

Data were presented as the mean ± standard deviation (SD). Statistical analyses were performed with GraphPad Prism 9.0. Group differences were analyzed by one-way analysis of variance (ANOVA) followed by Tukey’s post-hoc test for multiple comparisons. All groups were compared with the C group, which was denoted by asterisks (*). Differences were considered significant when the *P* values were <0.05 (*) and <0.01 (**). In addition, comparisons between the Cd group and intervention LYC+Cd group were performed and annotated separately, which were denoted by number signs (#). Differences were considered significant when the *P* values were <0.05 (#) and < 0.01 (##).

## Results

### LYC ameliorated Cd-induced growth retardation in broilers

**Fig. 1:**
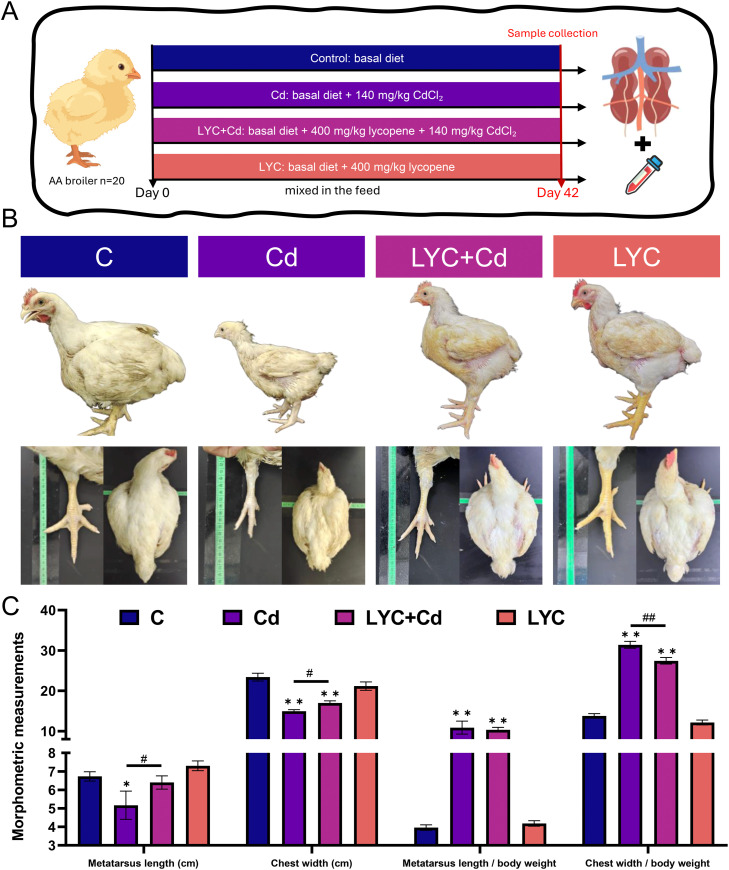
**LYC ameliorated Cd-induced growth retardation in broilers.** (A) Schematic diagram of establishment of animal model. (B) Animal clinical status. (C) Metatarsus length and chest width, each normalized to body weight. Data are presented as mean ± SD (*n* = 3 per group). Groups were compared by one-way ANOVA followed by Tukey’s post-hoc test. Comparisons versus the C group are indicated by asterisks: * *P* < 0.05, ** *P* < 0.01. Comparisons between the Cd and LYC+Cd groups are indicated by hash signs: # *P* < 0.05, ## *P* < 0.01.

A schematic representation of the overall experimental design was provided in Fig. 1A. Morphometric parameters (metatarsus length, chest width and their ratios to body weight), which can reflect the growth performance of broilers, were measured to indicate the skeletal growth and body conformation (Hakami et al., 2022). As shown in Fig. 1B, C, the metatarsus length and chest width of the Cd group were significantly reduced compared with the C group (*P* < 0.05, *P* < 0.01). The LYC+Cd group markedly restored these changes compared with the Cd group (*P* < 0.05). Moreover, the ratios of metatarsus length to body weight and chest width to body weight in the Cd group were noticeably increased compared with the C group (*P* < 0.01). The LYC+Cd group significantly attenuated the Cd induced rise of the ratios of metatarsus length/body weight and chest width/body weight (*P* < 0.01). These results indicated that Cd exposure induced growth retardation of broilers, and LYC supplementation significantly restored Cd-induced growth performance impairments.

### LYC attenuated Cd-induced renal structural and functional damage

**Fig. 2:**
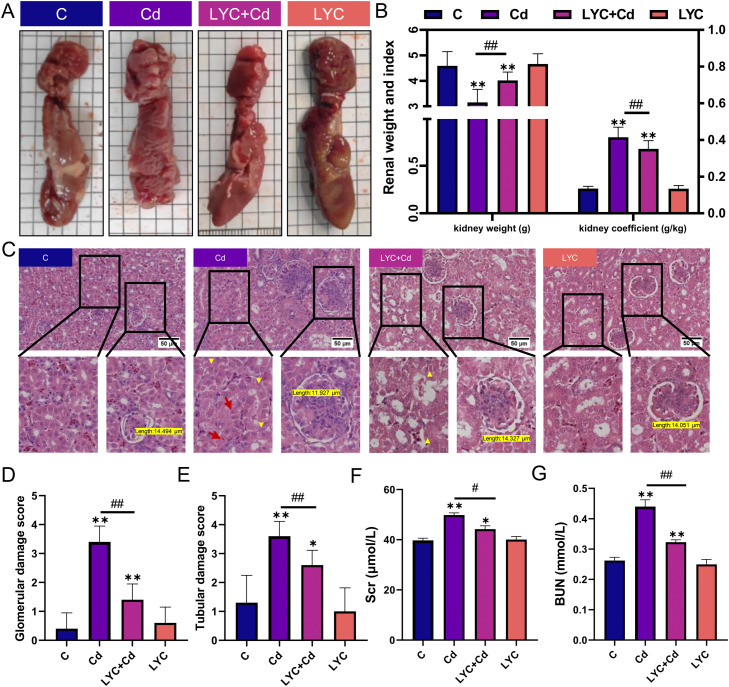
**LYC attenuated Cd-induced renal structural and functional damage.** (A) Appearance of kidney. (B) Kidney weight and kidney coefficient. (C) Kidney H&E staining. (D) Glomerular damage score. (E) Tubular damage score. (F) The serum content of SCr. (G) The serum content of BUN. In HE diagrams, red arrows indicated nuclear pyknosis of renal tubular epithelial cells, yellow triangles manifested that renal tubular epithelial cells lysis and shed. Data are presented as mean ± SD (*n* = 3 per group). Groups were compared by one-way ANOVA followed by Tukey’s post-hoc test. Comparisons versus the C group are indicated by asterisks: * *P* < 0.05, ** *P* < 0.01. Comparisons between the Cd and LYC+Cd groups are indicated by hash signs: # *P* < 0.05, ## *P* < 0.01.

As shown in Fig. 2A, clinical observations showed that the kidneys of broilers in the C and LYC groups were normal in shape, the Cd group appeared paler and smaller, and LYC+Cd group restored these changes compared with the Cd group. The Cd group showed a decrease in kidney weight and an increase in kidney coefficient compared with the C group, LYC+Cd group significantly reversed these changes in Fig. 2B (*P* < 0.01). As shown in Fig. 2C, histopathological examination showed normal glomerular and tubular structure in the C and LYC groups. Importantly, the Cd group exhibited marked glomerular swelling, cystic cavity narrowing and renal tubular epithelial cell necrosis, and the LYC+Cd group significantly alleviated the renal structural injury induced by Cd. As shown in Fig. 2D–E, semi-quantitative scoring showed that glomerular and tubular damage scores were significantly higher in the Cd group relative to the C group (*P* < 0.01). The LYC+Cd group reduced these histopathological scores relative to the Cd group (*P* < 0.01). As shown in Fig. 2F, G, the SCr and BUN were increased after Cd exposure and were clearly lowered by LYC supplementation (*P* < 0.05, *P* < 0.01). These results indicated that LYC mitigated renal structural and functional impairments induced by Cd.

### LYC alleviated Cd-induced renal oxidative stress and mitochondrial dysfunction

**Fig. 3:**
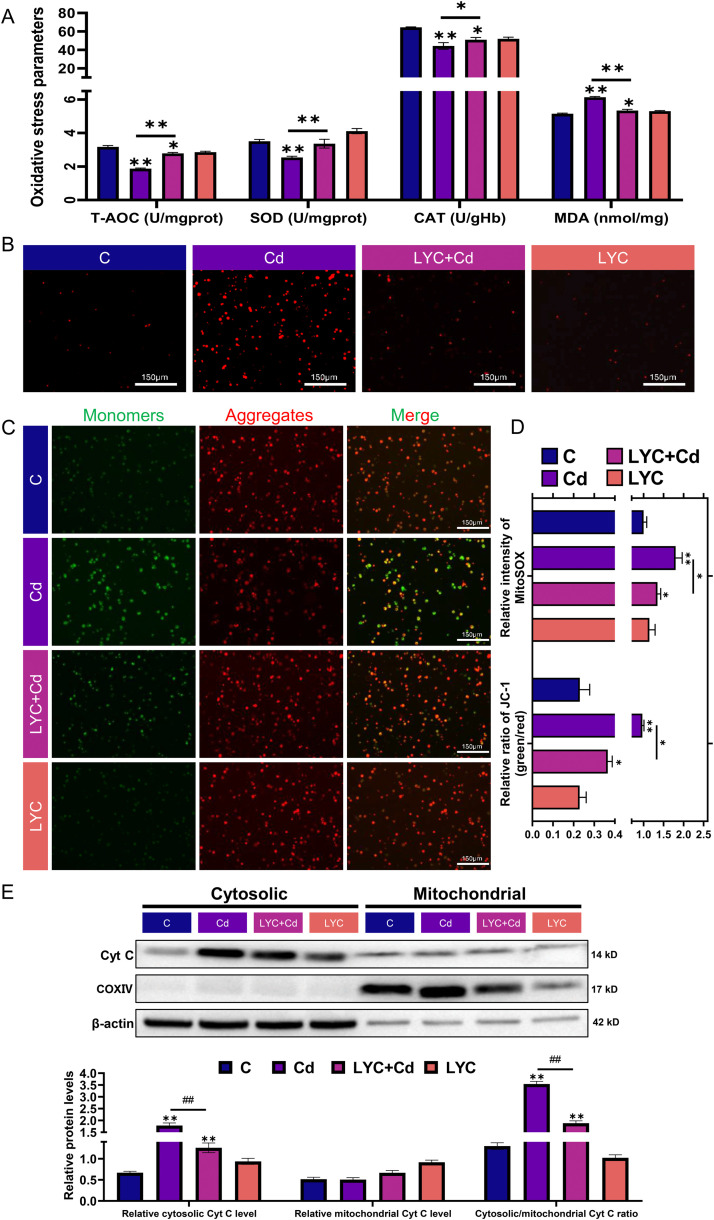
**LYC alleviated Cd-induced renal oxidative stress and mitochondrial dysfunction.** (A) Levels of oxidative stress-related parameters (T-AOC, SOD, CAT and MDA) in broiler kidney tissues measured by biochemical kits. (B) Representative fluorescence images of mitochondrial ROS staining (red) in broiler kidney tissues. (C) JC-1 staining of broiler kidney tissue showing ΔΨm. Red fluorescence indicates JC-1 aggregates (high ΔΨm) and green fluorescence indicates JC-1 monomers (depolarized mitochondria). (D) The corresponding quantitative analysis of MitoSOX fluorescence intensity and the red/green fluorescence ratio of JC-1. (E) Western blot analysis of Cyt c protein expression in both cytosolic and mitochondrial fractions of broiler kidney tissues. Data are presented as mean ± SD (*n* = 3 per group). Groups were compared by one-way ANOVA followed by Tukey’s post-hoc test. Comparisons versus the C group are indicated by asterisks: * *P* < 0.05, ** *P* < 0.01. Comparisons between the Cd and LYC+Cd groups are indicated by hash signs: # *P* < 0.05, ## *P* < 0.01.

As shown in Fig. 3A, renal T-AOC and the activities of CAT and SOD were decreased in the Cd group relative to the C group (*P* < 0.01). The LYC+Cd group evidently restored T-AOC and activities of SOD and CAT compared with the Cd group (*P* < 0.05, *P* < 0.01). The level of MDA was elevated in kidney of the Cd group compared with the C group (*P* < 0.01). The LYC+Cd group significantly decreased MDA levels relative to the Cd group (*P* < 0.01). As shown in Fig. 3B–D, MitoSOX fluorescence intensity was significantly elevated in the kidney tissue of the Cd group relative to the C group and markedly alleviated after LYC supplementation (*P* < 0.05, *P* < 0.01). Meanwhile, compared with the C group the ΔΨm in Cd group was significantly depolarized (*P* < 0.01) by JC-1 staining. LYC supplementation significantly restored these changes (*P* < 0.05). In parallel, Cd exposure caused a significant elevation of cytosolic Cyt C levels and a marked increase in the cytosolic/mitochondrial Cyt C protein ratio compared with the C group (*P* < 0.01), and LYC supplementation markedly reduced both cytosolic Cyt C and the Cyt C ratio (*P* < 0.01) (Fig. 3E). These results indicate that LYC can alleviate Cd-induced renal oxidative stress damage and mitochondrial dysfunction in broilers.

### LYC restored Cd-induced mitochondrial biogenesis and dynamics disorder

**Fig. 4:**
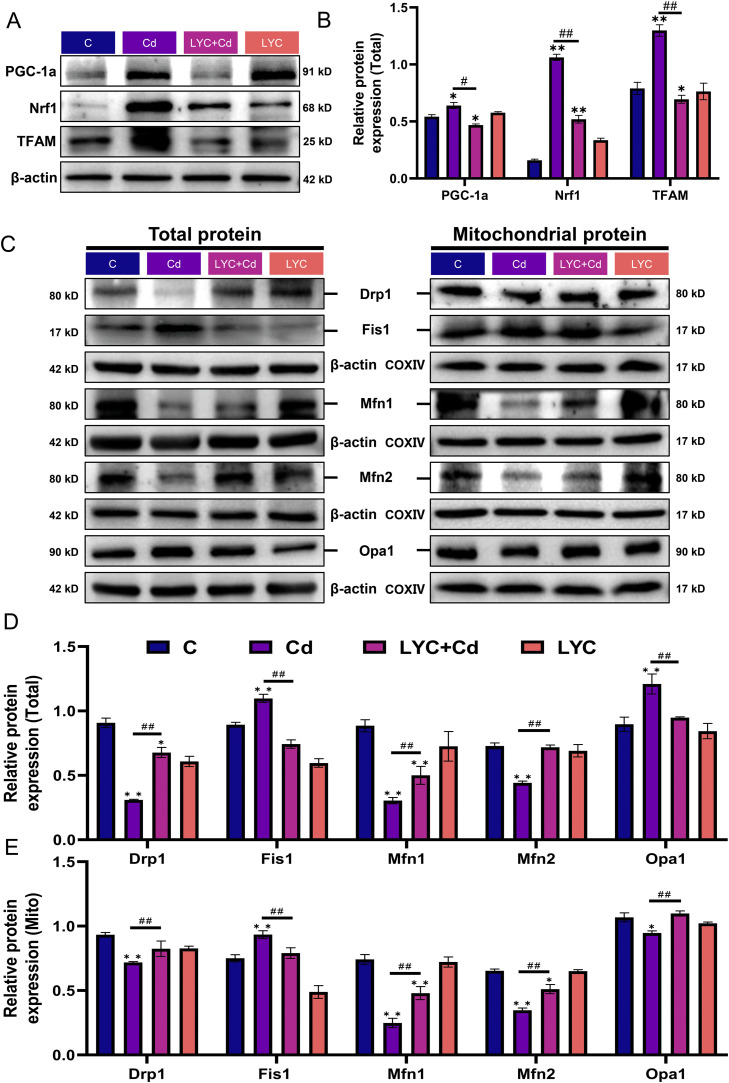
**LYC restored Cd-induced mitochondrial biogenesis and dynamics disorder.** (A–B) Western blot detected the expression of PGC-1α, Nrf1 and TFAM. (C–E) Western blot respectively detected the expression of Drp1, Fis1, Mfn1/2 and Opa1 in tissue and mitochondria. Data are presented as mean ± SD (*n* = 3 per group). Groups were compared by one-way ANOVA followed by Tukey’s post-hoc test. Comparisons versus the C group are indicated by asterisks: * *P* < 0.05, ** *P* < 0.01. Comparisons between the Cd and LYC+Cd groups are indicated by hash signs: # *P* < 0.05, ## *P* < 0.01.

As shown in Fig. 4A, B, the protein expression of mitochondrial biogenesis-related proteins (PGC-1α, Nrf1, TFAM) was upregulated in the Cd group relative to the C group (*P* < 0.05, *P* < 0.01). The LYC+Cd group markedly restored these proteins toward C group levels compared with the Cd group (*P* < 0.05, *P* < 0.01).

As shown in Fig. 4C–E, the protein expression of mitochondrial fusion markers (Mfn1 and Mfn2) was both decreased in tissue and mitochondria, whereas Opa1 was increased in tissues and decreased in mitochondria after Cd exposure (*P* < 0.05, *P* < 0.01). LYC supplementation rescued all these changes compared with the Cd group (*P* < 0.05, *P* < 0.01). Interestingly, Cd exposure decreased Drp1 expression and upregulated Fis1 expression in both tissue and mitochondrial fractions relative to the control group; however, LYC supplementation reversed these alterations (*P* < 0.01). Collectively, Cd exposure induced marked disturbances in mitochondrial biogenesis and dynamics, and these Cd-induced alterations were alleviated by LYC supplementation.

### LYC reversed Cd-induced mitophagy-related alterations in the kidney

**Fig. 5:**
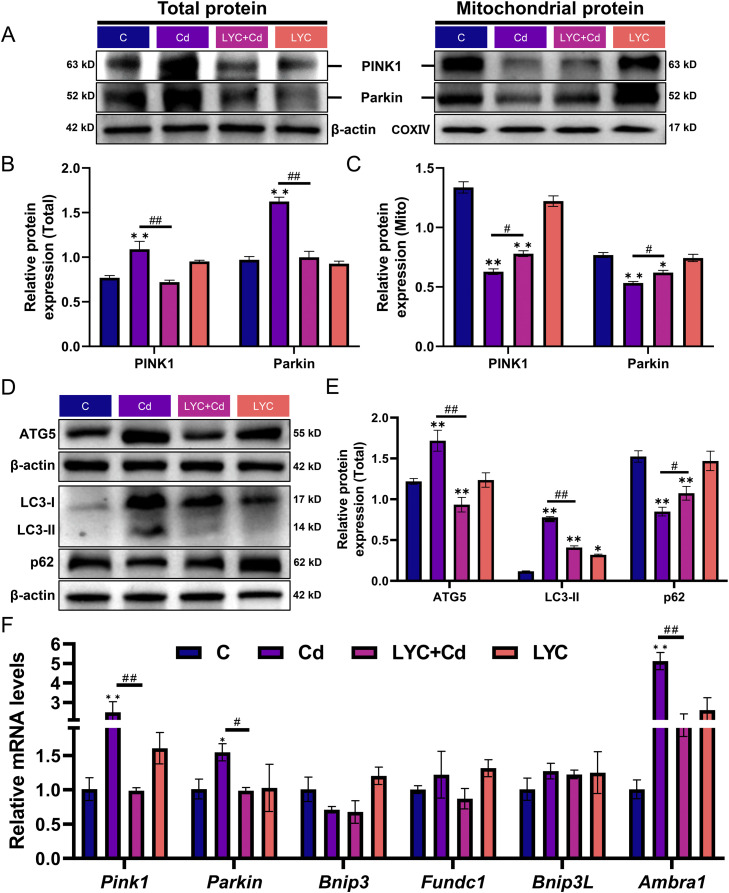
**LYC Reversed Cd-induced Mitophagy-related Alterations in the Kidney.** (A–C) Western blot respectively detected the expression of PINK1 and Parkin in tissue and mitochondria. (D–E) The protein expression of ATG5, LC3-Ⅱ and p62. (F) The mRNA expression of *Pink1, Parkin, Bnip3, Fundc1, Bnip3L* and *Ambra1*. Data are presented as mean ± SD (*n* = 3 per group). Groups were compared by one-way ANOVA followed by Tukey’s post-hoc test. Comparisons versus the C group are indicated by asterisks: * *P* < 0.05, ** *P* < 0.01. Comparisons between the Cd and LYC+Cd groups are indicated by hash signs: # *P* < 0.05, ## *P* < 0.01.

As shown in Fig. 5A–C, PINK1 and Parkin protein abundance in whole-kidney tissue was increased in the Cd group, whereas PINK1 and Parkin protein abundance were both decreased in mitochondria compared with C group after Cd exposure (*P* < 0.01). LYC supplementation restored all these changes toward the C group levels (*P* < 0.01). As shown in Fig. 5D, E, Cd exposure caused a clear increase in LC3-II and a decrease in p62 compared with the C group (*P* < 0.01). The LYC+Cd group restored these changes compared with the Cd group (*P* < 0.01). As shown in Fig. 5F, in whole-kidney tissue, among mitophagy-related genes, *Bnip3, Bnip3L* and *Fundc1* mRNA levels showed no significant changes, whereas the mRNA levels of *Ambra1, Pink1* and *Parkin* were significantly increased (*P* < 0.05, *P* < 0.01). LYC supplementation significantly attenuated these Cd-induced *Ambra1, Pink1* and *Parkin* changes (*P* < 0.05, *P* < 0.01). Collectively, these findings indicated that Cd induced mitophagy by the PINK1/Parkin axis, whereas LYC reversed these alterations. These data suggest that PINK1/Parkin-related mitophagy may be involved in the protective effects of LYC.

## Discussion

The kidney serves as both the primary accumulation site and excretion organ of Cd, rendering it highly susceptible to Cd attack. Increasing evidence indicated that mitochondrial dysfunction represents a key event in Cd-induced renal injury (Liu et al., 2019). MQC system perturbations play a pivotal role in the pathogenesis of Cd nephrotoxicity. Therefore, exploring agents with mitochondrial protective properties has become an important therapeutic strategy for mitigating Cd-triggered renal injury. In this study, dietary LYC significantly alleviated Cd-induced renal injury in broilers. LYC regulated mitochondrial dynamics-related factors and mitophagy-related markers to inhibit mitochondrial damage and maintain mitochondrial homeostasis.

Systemic Cd toxicity in poultry typically manifests as a significant decline in growth performance, which subsequently impairs overall production efficiency under both acute and chronic exposure conditions (Bilal and Erçağ, 2003). The growth and mineralization of the femur, tibia and metatarsus of broilers have been systematically compared, and identified the length and chest width of the metatarsus as sensitive morphological indicators for evaluating the longitudinal and transverse bone development of broilers, which can be used to evaluate the effects of pollutants on bone development (Han et al., 2015). Therefore, metatarsus length, chest width and their ratios to body weight were used as sensitive indices of skeletal growth and body conformation in broilers and were employed to assess growth performance in this study. Meanwhile, higher doses of Cd in the diet can cause poultry body weight, egg production, and bone mineralization defects and leg pathology (Ketta et al., 2021). In this study, Cd exposure reduced metatarsus length, chest width and their ratios to body weight in broilers; LYC co-treatment partially reversed these morphometric deficits and mitigated renal histopathological injury, indicating that LYC can mitigate the toxic effects of Cd in broilers.

The kidney is considered the primary reservoir for Cd bioaccumulation in poultry, and its structural integrity is the fundamental of systemic metabolic homeostasis. The accumulation of Cd in avian kidney tissues and its deleterious effects on growth and kidney function are well-established. Chronic Cd exposure leads to significantly higher Cd concentrations in the kidney than in blood, heart, liver, lung, or spleen by comparative biodistribution analyses in rodents, providing a pharmacokinetic basis for renal susceptibility (Young et al., 2019). In broiler models, dietary exposure to 0 mg/kg, 35 mg/kg or 70 mg/kg CdCl_2_ in diet for 90 days led to significant dose-dependent renal tubular epithelial cell swelling and abnormalities in renal function indicators such as SCr (Ge et al., 2019). Complementary evidence from a rat model demonstrated that sub-chronic or chronic oral Cd exposure produces characteristic renal pathology (including proximal tubular degeneration, oxidative stress and increased apoptotic markers) and induced renal dysfunction in a rat model (Liu et al., 2019). These studies collectively associate significant dose-dependent increases in serum SCr and BUN levels, and mitochondrial injury in renal tubular epithelial cells, supporting that Cd exposure directly damages kidney function. In this study, broiler models exposed to 140 mg/kg dietary Cd exhibited pronounced renal macroscopic lesions, increased histopathological and serum biomarkers (SCr and BUN), confirming that the Cd-induced kidney injury model in broilers was successfully established. Importantly, the partial normalization of kidney index and SCr/BUN by LYC revealed that LYC ameliorated both the structural and functional impairments induced by Cd in the kidney.

Mitochondrial damage, as the principal intracellular source of ROS and as a primary target of oxidative damage, is a major effect of Cd-induced nephrotoxicity. This damage is characterized by a loss of mitochondrial ΔΨm, impaired electron transport chain activity, and excessive ROS production. These defects collectively exacerbate oxidative stress, disrupt cellular energy metabolism, and ultimately culminate in tubular cell injury and renal dysfunction (Thévenod et al., 2020; Yan and Allen, 2021; Shi et al., 2025). Cd impairs electron transport chain function, promotes electron leakage and increases mitochondrial ROS. Conversely, excessive mitochondrial ROS attacks mitochondrial lipids, mtDNA, and respiratory proteins, causing membrane permeabilization, Cyt C release, and bioenergetic collapse, which further impairs electron transport and amplifies oxidative stress—creating a self-reinforcing injury loop (Branca et al., 2020). Exposure to Cd^2+^ (5–20 μM) rapidly induced Cyt C release from mitochondria into the cytosol in proximal tubular cells via Ca²⁺-dependent opening of the mitochondrial permeability transition pore, identifying mitochondrial membrane destabilization as an early and decisive event in Cd-induced renal apoptosis (Lee et al., 2005). Cd exposure induced marked mitochondrial dysfunction in the broiler kidney, as evidenced by a loss of mitochondrial ΔΨm and elevated ROS production. These findings suggest that Cd impairs the electron transport chain, thereby facilitating electron leakage, which serves as a primary driver for the subsequent uncontrolled ROS generation and mitochondrial membrane destabilization. Consequently, the excessive efflux of mitochondrial superoxide culminated in widespread renal oxidative damage; this was manifested by the marked accumulation of MDA and the exhaustion of antioxidant defenses, including T-AOC, SOD, and CAT. Moreover, both mitochondrial and cytosolic Cyt C were measured, and a significant elevation was found of cytosolic Cyt C after Cd exposure, directly supporting mitochondrial functional impairment and membrane permeabilization. LYC normalized these changes, suggesting that the nephroprotective effect of LYC includes maintenance of mitochondrial bioenergetic integrity and attenuation of mitochondria-derived oxidative markers. Overall, Cd triggers mitochondrial impairment and the production of excessive ROS, leading to renal oxidative stress, which in turn further exacerbates mitochondrial damage. LYC supplementation significantly attenuated these oxidative markers and restored the antioxidant defensive capacity, indicating that LYC effectively mitigates Cd-induced renal mitochondrial damage.

Mitochondrial homeostasis relies on the MQC system, a sophisticated network coordinating mitochondrial mass, morphology, and energy metabolism. Recent studies indicate that Cd exposure triggers organellar stress, thereby impairing the mitochondrial UPR and dysregulating the expression of proteins responsible for mitochondrial biogenesis, dynamics, and mitophagy (Ho and Shirakawa, 2022). Therefore, regulating the MQC system to maintain mitochondrial homeostasis has become a potential node in alleviating Cd-induced nephrotoxicity. Mitochondrial biogenesis is a compensatory process that increases mitochondrial mass and population to meet heightened ATP demands under stress, while ensuring the quality of the mitochondrial pool by replacing dysfunctional organelles with healthy counterparts. In cultured renal proximal tubular cells, short-term (24 h) acute exposure to 1, 10, 30 μM CdCl₂ resulted in increased mitochondrial DNA copy numbers and biogenesis-related signals (biogenesis markers) with fusion-related changes, suggesting that short-term exposure to low-to-medium doses activates biosynthesis (Nair et al., 2015). In human HK-2 renal proximal tubule cells exposed with CdCl₂ (0–60 μmol/L) for 24 h, mitochondrial ROS increased and ΔΨm decreased in a dose-dependent manner, accompanied by inhibition of respiratory chain complex III activity and a significant reduction in PGC-1α expression, indicating that Cd directly inhibits mitochondrial biogenesis (Yuan and Han, 2018). The regulation of mitochondrial biogenesis by Cd is dose-dependent, specifically, 35 mg/kg CdCl₂ evoked mitochondrial biogenesis through the transcriptional upregulation of SIRT1/SIRT3, PGC-1α, NRF1, and TFAM, whereas 70 mg/kg CdCl₂ inhibited mitochondrial biogenesis, thereby exacerbating mitochondrial damage (Ge et al., 2019). In this study, dietary exposure to CdCl₂ (140 mg/kg) stimulated mitochondrial biogenesis, as evidenced by the upregulated expression of biogenesis markers (PGC-1α, NRF1, and TFAM) in renal tissue. The sustained elevation of these factors suggests a compensatory response to Cd-induced mitochondrial depletion. LYC treatment effectively attenuated this biogenic response, likely because the mitigation of mitochondrial damage reduced the cellular demand for compensatory mitochondrial replenishment.

Mitochondrial dynamics, an orchestrated balance between mitochondrial fusion and fission, is fundamental for maintaining mitochondrial morphology, distribution, and functional integrity. Dietary CdCl₂ (20 mg/kg for 40 days) in pigs inhibited PI3K/AKT signaling pathway and disrupted mitochondrial dynamics in the hypothalamus, manifested as downregulated fusion markers (Mfn1/2, OPA1), while upregulated fission markers (Drp1, Mff), culminating in the induction of apoptosis and necroptosis (Chen et al., 2022). Cd exposure significantly downregulated the expression of fusion proteins (Mfn1/2) in both whole-tissue lysates and mitochondrial fractions. Although OPA1 expression was elevated in total protein lysates, a concomitant decrease was observed in the mitochondrial fraction. This discrepancy likely stems from stress-induced cleavage and redistribution of OPA1. Mitochondrial OPA1 exists in two primary isoforms: the long form (L-OPA1, membrane-anchored) and the short form (S-OPA1, soluble), and their relative abundance is subject to dynamic alteration under cellular stress. YME1L cleaves at steady state to maintain balance, while OMA1 is activated when ΔΨm is lost or oxidative stress occurs, rapidly cleaving L-OPA1 to become S-OPA1, resulting in a reduction of L-OPA1 on the inner mitochondrial membrane and promoting mitochondrial fragmentation. OPA1 cleavage and stress response by OMA1/YME1L have been reported in the literature (MacVicar and Langer, 2016; von der Malsburg et al., 2023). OPA1 proteolytic processing is a ΔΨm-sensitive, developmentally regulated and reversible response: in RA-differentiated H9c2 cardiomyoblasts CCCP-induced membrane depolarization provoked robust OMA1-dependent L-OPA1 cleavage to S-OPA1, resulting in mitochondrial fragmentation and altered cristae architecture; critically, this processing was reversible upon removal of the stimulus and was modulated by metabolic/differentiation state (Garcia et al., 2021). These findings provide a plausible explanation for the discrepancy between the elevated total OPA1 expression and its concurrent reduction within the mitochondrial fraction. Unexpectedly, total tissue and mitochondrial Drp1 protein were both decreased in Cd kidneys, while Fis1 was increased. This contrasts with many published reports showing Drp1 upregulation/mitochondrial recruitment after Cd exposure (Xu et al., 2013). This could stem from (i) temporal dynamics — early Cd exposure may transiently increase Drp1 activity/localization followed by proteolytic cleavage or degradation at later time points; (ii) post-translational modification (phosphorylation/SUMOylation) or protease-mediated cleavage could reduce detectable full-length Drp1 despite increased fission activity. Importantly, LYC normalized these markers, suggesting it stabilizes both mitochondrial renewal and network integrity.

Mitophagy is the selective removal pathway for damaged mitochondria and is central to the MQC system (Ge et al., 2020). Cd can activate PINK1/Parkin-mediated mitophagy via ROS signaling in kidney and other tissues; Antioxidant treatments, e.g. N-acetyl-L-cystine (NAC), often attenuate PINK1 accumulation in mitochondrial outer membrane and Parkin mitochondrial translocation, demonstrating that ROS acts as an upstream trigger. In a mouse model that short-term Cd exposure collapses mitochondrial ΔΨm, produces characteristic mitophagosomes and increases LC3-II and PINK1 levels, while pretreatment with NAC or Acetyl-L-carnitine attenuated ROS, preserved ΔΨm and mitochondrial mass and reduced PINK1/Parkin recruitment (Wei et al., 2014). In PC12 cells, Cd exposure activates AMPK, which in turn promotes PINK1 stabilization and Parkin recruitment to mitochondria, thereby initiating PINK1/Parkin-dependent mitophagy; genetic or pharmacologic inhibition of AMPK blunted mitophagy and enhanced NLRP3 activation, indicating the protective role of PINK1/Parkin-mediated mitophagy against Cd-induced mitochondrial injury (Wang et al., 2020). Previous studies have shown that PINK1 is rapidly stabilized on depolarized mitochondria and recruits Parkin from the cytoplasm to mitochondria, whereas Parkin detection in mitochondria-rich fractions may be faint because of weak mitochondrial association and instability during fractionation (Matsuda et al., 2010). Cd exposure causes substantial mitochondrial damage and depletion, which reduces the total number of recoverable mitochondria in isolated fractions and thus lowers overall mitochondrial protein yield. Therefore, the reduced mitochondrial abundance of PINK1 and Parkin does not indicate mitophagy suppression, but rather reflects two interconnected processes: the continuous lysosomal degradation of mitochondria-associated PINK1/Parkin during active mitophagy flux, and the global reduction in mitochondrial mass caused by Cd-induced injury. Therefore, the reduced PINK1 signal in the mitochondrial fraction observed in our study may reflect active turnover during mitophagic flux at the sampled time point. Because the current study evaluated a single experimental endpoint, the temporal dynamics of PINK1/Parkin redistribution could not be directly assessed. Future studies incorporating time-course analyses and mitophagy flux assays will be required to further clarify this phenomenon. Concurrently, the mRNA expression of *Pink1* and *Parkin* was upregulated. Mitophagy indicators showed LC3-II accumulation and decreased p62. Together, these findings indicated that the PINK1–Parkin mediated mitophagy was consistently activated in Cd-exposed kidneys. Mitophagy can also be mediated by specific receptors, including BNIP3/BNIP3L, FUNDC1, and AMBRA1 (Bai et al., 2021). In this study, the transcriptional activation of *Bnip3*/*Bnip3L*/*Fundc1* was not observed, except for *Ambra1*. These data collectively point to the activation of the PINK1/Parkin axis as a key event in Cd-induced mitophagy. It should be noted that the observed changes in LC3-II and p62 levels serve as biochemical indicators of mitophagic activity. While direct assessment of mitophagic flux is technically challenging in an *in vivo* broiler model, the coordinated alteration of PINK1/Parkin and mitophagic markers strongly suggests the activation of the mitophagic process. Importantly, LYC reduced tissue PINK1, Parkin and LC3-II accumulation and partially restored p62, and it restored mitochondrial PINK1/Parkin protein levels, indicating that LYC reversed mitophagy-related markers. This mitigative effect of LYC may stem from two mechanisms, depending on the site of action of LYC detoxification and the role of mitophagy. Firstly, LYC likely acts as an upstream mitochondrial protective shield by quenching ROS and reducing initial mitochondrial damage, thereby diminishing the biological demand for mitophagy to clear damaged mitochondria; secondly, LYC could reverse mitophagy-related marker changes by Cd, which itself acts as a pathological driver of renal injury. These results supported the hypothesis that the protective effects of LYC against Cd-induced renal injury may be associated with modulation of MQC-related factors and restoration of mitochondrial homeostasis.

Although our findings support an association between LYC protection and the restoration of MQC-related factors, we cannot exclude additional upstream regulators or parallel pathways. Future transcriptomic and metabolomic analyses will be necessary to provide an unbiased view of the molecular network underlying Cd toxicity and LYC protection. Whether LYC primarily functions through upstream damage prevention or downstream mitophagy regulation also remains to be further elucidated. Additionally, it should be noted that the Cd dose used in the present study was higher than the exposure levels typically encountered under field conditions, and therefore should be interpreted as an experimental challenge dose rather than a realistic production-level contamination scenario. Likewise, the LYC dose was selected primarily for mechanistic validation rather than direct field application. The purpose of this design was to establish a reproducible renal injury model and to evaluate the mechanistic protective effects of LYC under defined toxic stress. Future studies should further examine lower, environmentally relevant Cd concentrations and dose–response relationships of LYC to determine whether the protective effects observed here can be translated to more practical supplementation strategies in poultry production.

## Conclusion

Dietary LYC could alleviate Cd-induced renal injury in broilers, potentially through modulating MQC-related signaling pathways to maintain mitochondrial structural and functional integrity. LYC may be a promising feed additive candidate to reduce Cd nephrotoxicity in poultry production due to its protective effects associated with mitochondrial homeostasis regulation. Further mechanistic and translational studies will be important for validating LYC as a practical intervention.

## Funding

This work was supported by the Joint Fund of the Key Project of Henan Province Science and Technology Research and Development Plan (252301420012, 245200810018), the Key Project of Henan Province Science and Technology Research (262102311264), the 10.13039/501100001809National Natural Science Foundation of China (32102739), and Research and Innovation Team of Henan vocational college of agriculture in 2025 (2505TDLX05).

## CRediT authorship contribution statement

**Haodong Wang:** Writing – review & editing, Writing – original draft, Visualization, Validation, Supervision, Software, Resources, Project administration, Methodology, Investigation, Funding acquisition, Formal analysis, Data curation, Conceptualization. **Zhi Lu:** Software, Resources, Methodology, Formal analysis. **Gongyin Chen:** Writing – review & editing, Validation. **Xuebing Wang:** Methodology, Investigation. **Xu Yang:** Supervision, Project administration, Funding acquisition, Conceptualization. **Cong Zhang:** Writing – review & editing, Supervision, Project administration, Funding acquisition, Conceptualization.

## Disclosures

The authors declare that they have no known competing financial interests or personal relationships that could have appeared to influence the work reported in this paper.

## Appendix Supplementary materials

Image, application 1

## Footnotes

Supplementary material associated with this article can be found, in the online version, at doi:10.1016/j.psj.2026.107251.
