# Supplementary material for: Lycopene-mediated mitigation of cadmium-induced nephrotoxicity in broilers is associated with restoring mitochondrial homeostasis
Source: Poult Sci. 2026 Jun 10;105(9):107251. doi: 10.1016/j.psj.2026.107251 (PMC13311819; doi:10.1016/j.psj.2026.107251)
Supplement: Supplementary file 1 [file mmc1.docx]

**Table S1 Primer sequence**

| Gene | Forward Primer(5’→3’) | Reverse Primer(5’→3’) | Product length | NCBI accession No. |
| --- | --- | --- | --- | --- |
| β-actin | GCCCTGGCACCTAGCACAATG | CTCCTGCTTGCTGATCCACATCTG | 129 | [NM_205518.2](https://www.ncbi.nlm.nih.gov/entrez/viewer.fcgi?db=nucleotide&id=2124747379) |
| PINK1 | ACCCTGTGCCAGTATTTGAG | CTTCAGGTCTCTGTGTGCTATC | 123 | NM_001389481.2 |
| Parkin | TTGGCTACTCCCTCCCGTGTG | ACGCACTCCTCCGCACCATAG | 124 | XM_419615.8 |
| BNIP3 | TTCAAACACCCCAGACGCAC | TCCCAATGTAAATCCCGAGTCC | 154 | XM_421829.8 |
| BNIP3L | GCTTTGGGACTAGGCATCTAC | ACGAGACAGAAACACCTTCAC | 120 | NM_001030885.3 |
| FUNDCI | TGGAATCGTTTGTTTGGCCG | AAAATCCCGCACACCAGCC | 103 | NM_001276363.2 |
| AMBRA1 | TGGGAGGGCAAGAAAGTTGA | GAAGGTCACACACCAGGGAG | 186 | XM_046919523.1 |

| **Protein** | **Dilution ratio** | **Conpany** | **Product Code** |
| --- | --- | --- | --- |
| β-actin | 1: 5000 | Servicebio | GB15001 |
| COXⅣ | 1:5000 | Proteintech | 66110-1-Ig |
| Cyt C | 1:1000 | Servicebio | GB11080 |
| PCG-1a | 1:1000 | Servicebio | GB11912 |
| TFAM | 1:1000 | ABclonal | A13552 |
| Nrf1 | 1:1000 | Servicebio | GB11351 |
| Mfn1 | 1:1000 | ABclonal | A9880 |
| Mfn2 | 1:1000 | ABclonal | AB606 |
| Opa1 | 1: 1000 | Servicebio | GB111728 |
| Drp1 | 1:1000 | Wanleibio | WL03028 |
| Fis1 | 1:1000 | ABclonal | A5821 |
| PINK1 | 1:1000 | Wanleibio | WL04963 |
| Parkin | 1:4000 | Proteintech | Ag5179 |
| LC3B | 1:1000 | ABclonal | A19665 |
| p62 | 1:1000 | Wanleibio | WL02385 |
| ATG5 | 1:2000 | Wanleibio | WL02411 |

**Table S2 List of primary antibodies**
